# Effects of Taurine on Primary Metabolism and Transcription in a Coral *Symbiodinium* sp.

**DOI:** 10.3389/fmicb.2022.797688

**Published:** 2022-07-11

**Authors:** Aiyou Huang, Hejing Shi, Ruoxuan Cui, Xiaoni Cai, Zhenyu Xie

**Affiliations:** ^1^State Key Laboratory of Marine Resource Utilization in the South China Sea, Hainan University, Haikou, China; ^2^Laboratory of Development and Utilization of Marine Microbial Resource, Hainan University, Haikou, China; ^3^Key Laboratory of Tropical Hydrobiology and Biotechnology of Hainan Province, Haikou, China; ^4^College of Marine Sciences, Hainan University, Haikou, China

**Keywords:** coral reefs, host release factors, taurine, symbiosis, *Symbiodinium*

## Abstract

Coral reefs belong to the marine ecosystems and host the richest biodiversity of marine organisms. Coral reefs are formed as a result of the symbiotic relationship between the host coral animal and photosynthetic dinoflagellates, namely *Symbiodinium* sp. Coral animals induce the release of carbon fixation products of symbiotic *Symbiodinium* sp. through secreting host release factors (HRFs) such as taurine. To study the potential effect of taurine on photosynthesis and release of carbon fixation products of *Symbiodinium* sp., we compared the growth of *Symbiodinium* sp. under control and taurine-stimulated conditions. Photosynthesis parameters were detected to monitor the photosynthetic efficiency. Biomass and the contents of total soluble sugar, total insoluble sugar, total protein, total lipids, chlorophyll a were analyzed. Metabolome and transcriptome analyses were performed to analyze the potential effect of taurine on primary metabolism and mRNA transcription. The results revealed that taurine significantly increased the growth, photosynthesis efficiency, total soluble sugar, chlorophyll a, and chlorophyll b and free amino acid content of *Symbiodinium* sp. while decreased the content of total insoluble sugar. Results of metabolome and transcriptome analyses suggested that taurine might affect metabolic pathways in *Symbiodinium* sp. by altering the permeability of the algal cell membrane, diverting photosynthetically fixed carbon from storage compounds to translocated compounds, releasing a signal of low concentrations of nitrogen to initiate a series of response mechanisms, and controlling the density of *Symbiodinium* sp. through the quorum sensing effect. These results help to explore how corals control carbon metabolism in *Symbiodinium* sp. and to provide theoretical guidance for furthering our understanding of *Symbiodinium* sp. biology and coral-algal symbiosis.

## Introduction

Coral reefs play important roles in the marine ecosystem (Yu, 2018). Coral reefs are mainly distributed in tropical and subtropical waters and are the most diverse among all marine ecosystems in the world. Therefore, coral reefs are also known as “oasis in the blue desert” or “tropical rain forest in the ocean.” Coral reefs provide humans with huge social, economic, and ecological benefits. First, coral reefs provide habitat for more than a quarter of all marine fish species and a large number of shellfish, crustaceans, and seaweeds, thus providing nutritional seafood to humans (Plaisance et al., 2011; Jury et al., 2019). Second, the colors of living corals in the ocean enhance the esthetic value of oceans and contribute to tourism, thus boosting global economies. Third, coral reefs can protect the coastline by resisting wind and erosion, decomposing harmful substances, and purifying seawater resources, thus acting as a buffer against environmental phenomena (Shi et al., 2011; Wu et al., 2011). However, coral reef ecosystems are facing severe threats due to unwanted human activities and the deterioration of the natural environment. In order to protect the coral reefs, it is necessary to have an in-depth understanding of their formation processes and mechanisms.

To the best of our knowledge, the formation of coral reefs depends to a great extent on the symbiotic relationship between coral animals and their symbiotic photosynthetic dinoflagellates namely *Symbiodinium* sp. (Muscatine and Cernichiari, 1969). The reef-building coral polyps and *Symbiodinium* sp. symbiont absorb carbon dioxide from seawater and convert it into calcium carbonate, which is secreted outward to form an exoskeleton. After 1,000 of years, the coral remains have accumulated and eventually become coral reefs. In this mutually beneficial symbiotic relationship, coral polyps provide shelter for the *Symbiodinium* sp., and their metabolic wastes, such as carbon dioxide, phosphate, and nitrate, can promote photosynthesis in *Symbiodinium* sp., while *Symbiodinium* sp. absorb wastes from corals metabolism and provide organic photosynthates and high concentrations of oxygen to corals, thereby accelerating respiration and calcification rates in coral (Muscatine and Porter, 1977; Liu et al., 2018). This close symbiosis allows them to live in the tropical-subtropical shallow waters where nutrients are limited. Therefore, studying the interaction mechanism of corals and *Symbiodinium* sp., including the mechanism of material exchange, will help in understanding the formation processes and mechanisms of the coral reef. However, the symbiotic relationship between corals and *Symbiodinium* sp. is not fully understood, i.e., it is unclear how corals induce the production of photosynthetic carbon products in *Symbiodinium* sp. for their growth.

Carbon fixation products of photosynthesis in *Symbiodinium* sp. are used for three main purposes: the growth of *Symbiodinium* sp., long-term storage of photosynthetic products in the form of carbohydrates or fatty acids (Hillyer et al., 2017), and formation of coral secretions (Bertucci et al., 2013). For coral symbiotic *Symbiodinium* sp., the fixation products of photosynthesis primarily help in the formation of coral secretions such as calcium carbonate (Bertucci et al., 2013). The host can effectively control the symbiotic *Symbiodinium* sp. to transfer most of its fixed carbon to the host (Davy and Cook, 2001). Previous studies suggested that in symbionts, hosts such as corals control the direction of carbon fixation products of symbiotic *Symbiodinium* sp. through the release of host release factors (HRFs) such as taurine (Grant et al., 2004). The addition of coral polyp cell homogenate to the culture of free-living *Symbiodinium* sp. significantly enhances the release of photosynthetic carbon fixation products by 2–3 times, indicating that HRFs, which is still active *in vitro*, can direct the flow of carbon products from free-living *Symbiodinium* sp. to the host. These HRFs are mainly free amino acids, such as taurine (Gates et al., 1995; Wang and Douglas, 1997). However, the mechanism of action of these factors has not yet been elucidated.

In this study, to explore the potential effect of taurine on photosynthesis and the direction of photosynthetic carbon products in *Symbiodinium* sp., we compared the growth of *Symbiodinium* sp. under control and taurine-stimulated conditions. Photosynthesis parameters were detected to monitor the photosynthetic efficiency. Biomass and the contents of total soluble sugar, total insoluble sugar, total protein, total lipids, chlorophyll a were analyzed to learn the probable influence of taurine on cellular components. Metabolome and transcriptome analyses were conducted to determine the effect of taurine on primary carbon metabolism and transcription in *Symbiodinium* sp. The results will help to explore the mechanism by which corals control the flow of carbon products produced by *Symbiodinium* sp. Moreover, we hoped to provide a theoretical explanation for advancing our understanding of *Symbiodinium* sp. biology and coral-*Symbiodinium* sp. symbiosis, and provide theoretical basis for protecting the coral reefs.

## Materials and Methods

### Symbiotic *Symbiodinium* sp. Isolation and Identification

*Symbiodinium* sp. were isolated from the stony coral *Pocillopora damicornis*, as described previously (Shaw et al., 2012; Zhou et al., 2018). Briefly, the stony coral *P. damicornis* was collected from the coral reef in Wenchang, Hainan Province, China, and maintained in filtered seawater at 26°C. A piece of coral tissue was removed randomly from each of three different *P. damicornis* colonies, and *Symbiodinium* sp. were then isolated from the three pooled tissues. Immediately after removal, the tissues were homogenized in 0.22 μm filtered seawater using a rotary-blade tissue homogenizer, filtered through 37 μm nylon mesh to remove animal tissues, followed by two rounds of centrifugation at 1,700 × *g* for 1 min. The pellet was resuspended in 0.22 μm filtered seawater after each centrifugation. Then, the resuspended sample was plated on a plate made with F/2 medium plus 1.5% agarose. Single brown colonies on the plate were taken and put into a 50 ml triangle bottle filled with 20 ml F/2 medium. The procedure was repeated three times to purify the strain. The F/2 medium was made with inorganic nutrients (containing 75 mg L^−1^ NaNO_3_, 5 mg L^−1^ NaH_2_PO_4_·2H_2_O, 3.2 mg L^−1^ FeCl_3_·6H_2_O, 1.8 mg L^−1^ EDTANa_2_, and 20 mg L^−1^ Na_2_SiO_3_·9H_2_O), trace elements, and vitamins (filter-sterilized).

To identify the isolated microalgae, a total of 1 ml microalgal biomass was harvested by centrifugation at 5,000 × *g* for 3–5 min, the resulting pellet was dissolved in 50 μl of lysis buffer (1 M Tris–HCl, pH 8.0; 0.5 M EDTA, pH 8.0), and then incubated at 95°C for 5 min. The supernatant obtained after centrifugation was used as a template for the PCR amplification of microalgal *18S rRNA* gene fragments. The total reaction volume was 25 μl, which contained 1 μl of template, 1 μl of 18S-F (CCTGGTTGATCCTGCCAG) and 1 μl of 18S-R (TTGATCCTTCTGCAGGTTCA) universal primers, 12.5 μl 2× Rapid Taq Master Mix (Vazyme, China), and 9.5 μl ddH_2_O. PCR conditions were set as follows: initial denaturation at 94°C for 5 min, followed by 30 cycles of denaturation at 94°C for 30 s, annealing at 55°C for 30 s, and extension at 72°C for 1 min, and final extension at 72°C for 10 min. The amplicons were sent to BGI Tech (Shenzhen, China) for sequencing.

### Cell Culture and Treatments

Algal cells were cultured in sterilized seawater supplemented with F/2 medium (Guillard, 1975). Cultures were incubated under cool white fluorescent light (~100 μmol m^−2^ s^−1^) at ~26°C and a 12:12 light:dark cycle. For treatment with low concentrations of taurine (LT), cells were treated with taurine at a final concentration of 10 μM, and an equal amount of NO_3_^−^ was added to control cells (LC). For treatment with high concentrations of taurine (HT), cells were treated with taurine at a final concentration of 1 mM, and an equal amount of NO_3_^−^ was added to control cells (HC). Each treatment was administered in triplicate sets in 1 L flanks. To monitor cell growth, the flank was stirred vigorously to suspend the cells and 5 ml of culture was sampled. A UV/visible spectrophotometer (UV-1800, Shimadzu, Japan) was used to measure the absorbance at 680 nm (A680 nm). When there were significant differences in growth (LT vs. LC and HT vs. HC) and photosynthesis performance (HT vs. HC) between treatments, algal cells were allowed to settle at the bottom of the flasks, and the supernatant was siphoned out. Cell pellets were collected by centrifugation at 10,000 × *g* for 10 min. Subsequently, the pellets were frozen in liquid nitrogen and stored at −80°C.

### Determination of Photosynthetic Performance

For photosynthetic performance measuring, cells were cultured under HC, HT, and NN (neither NO_3_^−^ nor taurine was added). Each treatment was administered in triplicate sets in 1 L flanks. The flank was stirred vigorously to suspend the cells and 5 ml of culture was sampled at days 1, 2, 3, 5, 7, and 9 for subsequent PSII activity detection with water-PAM (Falkowski and Raven, 2013). Act-Light, which was turned on immediately after sampling from the culture light intensity for measuring actual photon yield (YII), was set to 100 μmol m^−2^ s^−1^. After measuring YII, samples were incubated in darkness for 20 min. After dark-adaptation, the maximum photon yield (Fv/Fm) was measured under shading conditions.

### Metabolome Analysis

For metabolome analysis, each group included five biological replications. Metabolome analysis followed the methods described by Huang et al. (2015). Briefly, 50 mg of freeze-dried algal powder was resuspended in 1 ml of 50: 50 (v/v) methanol:water solution, vortexed for 1 min, and then centrifuged at 13,000 × *g* for 5 min. Supernatants (700 μl) were recovered, lyophilized, and resuspended in 450 μl of distilled water. Then, 50 μl DSS (2,2-dimethyl-2-silapentane-5-sulfonate) standard solution (Anachro, Canada) was added, and the solution was mixed by vortexing. The samples were transferred to 5-mm nuclear magnetic resonance (NMR) tubes (Norell, Landisville, NJ, United States), and spectra were collected using a Bruker AV III 600 MHz spectrometer. The first increment of the 2D-1H, 1H-NOESY pulse sequence was used for the acquisition of 1H-NMR data and for suppressing the solvent signal. Each run consisted of 100 ms mixing and 990 ms pre-saturation times (~80 Hz gammaB1), and the spectra were collected after obtaining a total of 128 scans in a 15 min period at 25°C. Fourier transformation was performed with the free induction decay (FID) signals in a Chenomx NMR Suite, version 8.3 (Chenomx Inc., Edmonton, Canada), and the baseline was corrected. DSS was used as the internal standard, and all spectra were referenced and analyzed against the Chenomx compound library.

### Transcriptome Analysis

For transcriptome analysis, each treatment was conducted in three replications. Total RNA was extracted using TRIzol reagent (Invitrogen, Carlsbad, CA, United States) according to the manufacturer’s instructions. High-quality total RNA (OD 260/280 = 1.8 ~ 2.2, OD 260/230 ≥ 2.0, RIN ≥ 6.5, 28S: 18S ≥ 1.0, > 2 μg) was used to construct cDNA libraries for high-throughput RNA sequencing. One microgram of total RNA was used to construct an RNA-seq transcriptome library using the TruSeqTM RNA sample preparation Kit from Illumina (San Diego, CA, United States) following the manufacturer’s instructions. Then, cDNA libraries were selected for cDNA target fragments of 200–300 base pairs (bp) in 2% low-range ultra agarose and then amplified using Phusion DNA polymerase (New England Biolabs (Beijing) LTD, Beijing). The amplified cDNA libraries were loaded into a NovaSeq 6000 sequencing system (Illumina).

To generate clean reads, raw sequence reads were trimmed using SeqPrep,^1^ and the quality of the raw reads was controlled using Sickle^2^ with default parameters. The clean reads were annotated according to Gene Ontology (GO), Kyoto Encyclopedia of Genes and Genomes (KEGG), Clusters of Orthologous Groups of proteins (COG), the NCBI non-redundant protein sequences database (NR), Swiss-Prot, and Pfam databases. The mapped reads were then normalized using the RPKM (reads per kb per million) method for the identification of differentially expressed genes (DEGs). Abundant genes were quantified using RSEM^3^ (Li and Dewey, 2011). Differential gene expression was determined using the “edgeR” package in R^4^ (Robinson et al., 2010) based on the following threshold parameters: log_2_ (fold-change [|FC|]) > 2 and value of *p* < 0.05. Functional annotation and enrichment analyses were performed and classified using the GO and KEGG databases. Detail parameters for bioinformatic pipelines are supplied in Supplementary Data 1.

### Cell Component Analysis

#### Total Lipid Analysis

Total lipids were extracted as the methods described by Bligh and Dyer (1959) and Huang et al. (2019) with minor modifications. Briefly, ~20 mg freeze-dried cells were suspended with 1 ml of chloroform: methanol = 1: 1 (v/v) and stirred vigorously for 5 min, after which 0.3 ml of 0.2 M H_3_PO_4_ (containing 1 M KCl) was added and mixed, followed by centrifugation at 5,000 × *g* for 5 min, then the solvent phase was recovered. This process was repeated three times. The solvent phases were combined, washed three times with distilled water, and then evaporated at ~20°C under a ventilated fume hood.

#### Total Soluble and Total Insoluble Sugar Analysis

Total soluble sugar content was determined as the methods described previously (Huan et al., 2014; Brányiková et al., 2015) with minor modifications. Cells were harvested and dried as described above (Section “Cell Culture and Treatments”). Then, ~5 mg freeze-dried cells were resuspended with 1 ml of 80% (v/v) ethanol, stirred vigorously for 5 min, and incubated at 68°C for 15 min. The supernatant was collected after centrifugation at 8,000 × *g* for 5 min. This process was repeated three times, and the supernatants were merged and evaporated at 85°C to a volume of ~0.3 ml, made up to 1 ml with distilled water, and used for the soluble sugar analysis. To hydrolyze insoluble sugar, 1 ml of 30% perchloric acid was added to the sediment and stirred for 15 min at room temperature (~25°C) and centrifuged at 8,000 × *g* for 5 min. The supernatant was collected. This extraction was repeated three times and the supernatants were merged and made up to 3 ml with 30% perchloric acid. To determine the content of sugar, 0.2 ml samples were added to 1 ml of anthrone solution [2 g anthrone in 1 L 72% (v/v) H_2_SO_4_] and mixed rapidly. The mixtures were incubated in a water bath at 100°C for 8 min, cooled to room temperature, and the absorbance at 625 nm was measured. The soluble sugar content was determined according to a standard curve carried out simultaneously using glucose, while the starch contents were calculated by multiplying the value by 0.9.

#### Total Protein Analysis

Total protein content was determined as the methods described by Huang et al. (2019). Briefly, ~5 mg of freeze-dried cells was hydrolyzed in 100 μl of 1 M NaOH at 80°C for 10 min. Following this, 900 μl H_2_O was added. The mixture was centrifuged at 12,000 *g* for 15 min and the supernatant was collected. This process was repeated three times, and all the resulted supernatants were merged. Protein concentration was then measured by a bicinchoninic acid protein assay kit using bovine serum albumin as standard.

#### Pigment Analysis

Briefly, cell pellets were mixed with 8 ml of methanol for 24 h at 4°C. Extracts were centrifuged at 5,000 × *g* for 10 min, and supernatants were recovered. Chlorophyll a was determined by detecting the absorbance levels at 665 and 652 nm and calculated using the equations described previously (Arnon, 1949). CDW was obtained after drying and weighing an equal amount of cell pellet to that used in the extraction.

### Statistical Analysis

For metabolomics, each treatment was conducted with five replications. For other analysis, each treatment was conducted in three replications. Student’s *t*-test was used to detect the significance of differences among samples, and the statistical significance level was set at value of *p* < 0.05. The results were presented as the mean ± standard deviation, and error bars were used to express the standard deviation.

## Results

### Effect of Taurine on the Growth of *Symbiodinium* sp.

**Figure 1:**
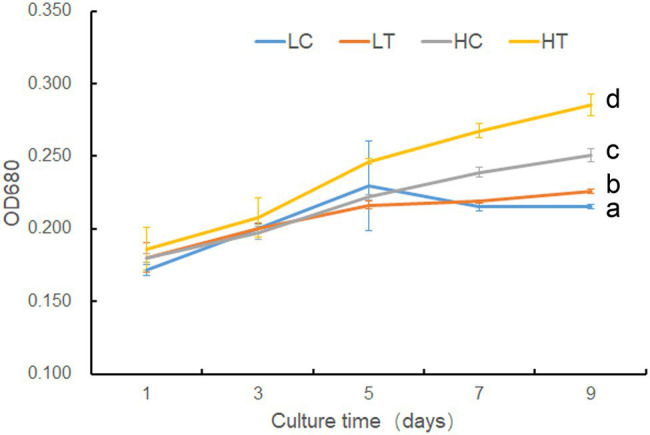
Growth of *Symbiodinium* sp. under different taurine concentrations. LT, treatment with low concentrations (10 μl) of taurine (LT); LC an equal amount of NO_3_^−^ was added to low concentrations control cells (LC); HT, treatment with high concentrations (1 mM) of taurine (HT); HC an equal amount of NO^3−^ was added to high concentrations control cells (HC). Data points are the means of triplicates and error bars represent the standard deviation. Letters a–d represent significant differences (value of *p* <0.05) between groups evaluated by Student’s *t*-test.

Based on 18S rDNA sequencing, these *Symbiodinium* sp. were identified as *Symbiodinium* sp., a ubiquitous endosymbiont associated with corals. The sequence data were available as Supplementary Data 2. To verify the potential effect of taurine addition on the growth of *Symbiodinium* sp., we compared the growth rate of *Symbiodinium* sp. under LC, LT, HC, and HT conditions. The results showed there was no significant difference (value of *p* > 0.05) in the growth rate of *Symbiodinium* sp. between LC and LT groups until day 9, at which time the growth rate of LT was higher (value of *p* < 0.05) than that of LC (Figure 1). However, taurine at high concentrations (HT/HC) significantly increased (value of *p* < 0.05) the growth rate of *Symbiodinium* sp. since day 3 (Figure 1).

### Photosynthetic Performance in *Symbiodinium* sp. Under HC and HT Conditions

**Figure 2:**
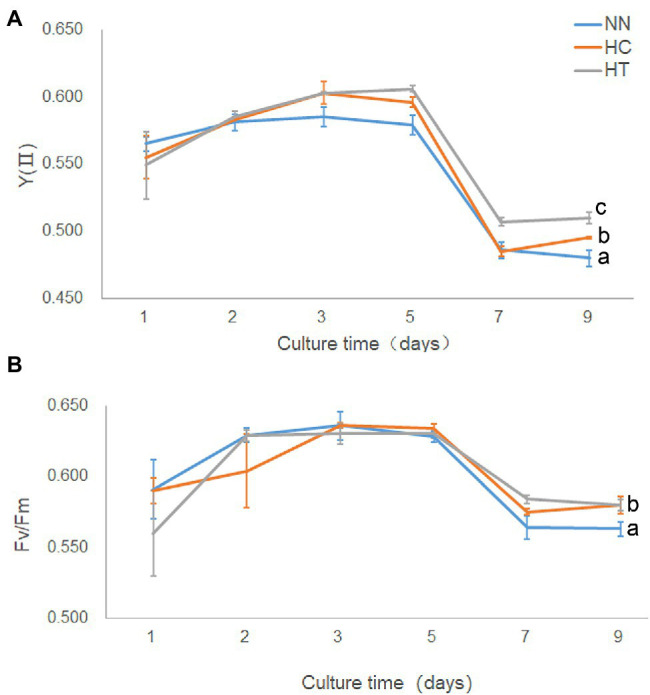
Photosynthetic performance and growth in *Symbiodinium* sp. under HC and HT and NN conditions. X-axis, culture times; Y-axis, YII **(A)** and Fv/Fm **(B)**. Data points are the means of triplicates and error bars represent the standard deviation. Letters a–c represent significant differences (value of *p* < 0.05) between groups evaluated by Student’s *t*-test.

Since the growth rate was highest in HT, followed by HC, and lowest in NN (Supplementary Figure S1), the photosynthetic performance was compared between these groups. On day 9 after inoculation, YII was highest in HT, followed by HC, and lowest in NN, indicating that taurine could increase actual photosynthetic efficiency in *Symbiodinium* sp., while N-limited condition decreased (value of *p* < 0.05) it (Figure 2A). There were no significant differences (value of *p* > 0.05) in Fv/Fm among HT, HC, and NN in early state of cultivation (Figure 2B). While on day 9, Fv/Fm in NN was significantly lower (value of *p* < 0.05) than that of HT and HC, indicating that N-limited condition influence the max photosynthetic efficiency in *Symbiodinium* sp.

### Result of Metabolome Analysis

**Figure 3:**
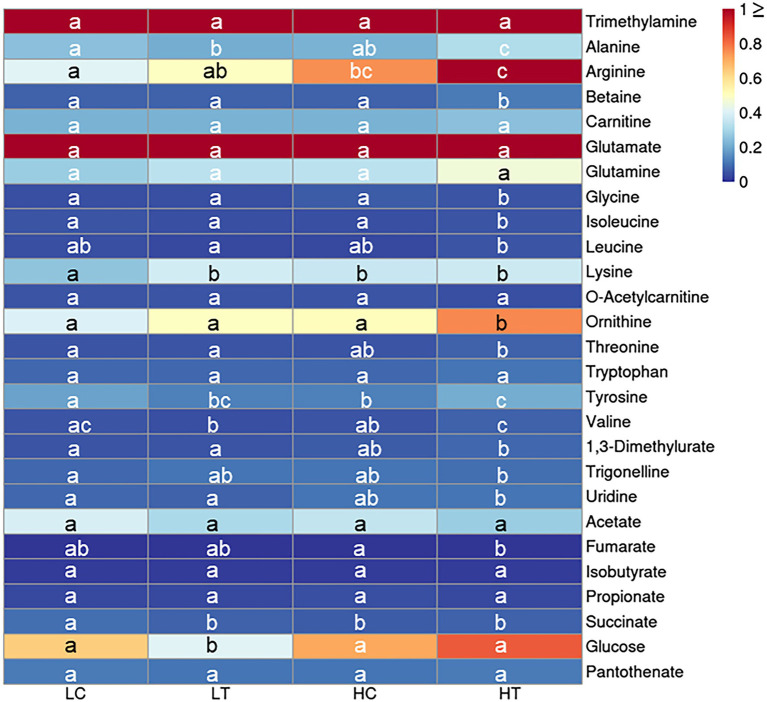
Heatmap for key cellular metabolites detected by NMR in *Symbiodinium* sp. under LC, LT, HC, and HT and NN conditions. Letters a–c represent significant differences (value of *p* < 0.05) between groups.

The metabolome was compared between algal cells under different culture conditions (LC, LT, HC, and HT). A total of 27 differently expressed metabolites were identified (Supplementary Table S1), including amines (*n* = 1), amino acids (*n* = 16), food and drug compounds (*n* = 2), nucleic acid components (*n* = 1), organic acids (*n* = 5), sugar (*n* = 1), and vitamin/cofactors (*n* = 1). The regulation patterns of most metabolites were similar in HT compared to the other three groups (i.e., HT/HC, HT/LN, and HT/LC), indicating that a high concentration (1 mM) of taurine can significantly affect the primary metabolism of *Symbiodinium* sp. (Figure 3). Of the 16 amino acids detected, seven were significantly increased in HT/HC, 10 were significantly increased in HT/LT, and eight were significantly increased in HT/LC groups, respectively (*p* < 0.05 for all). The contents of organic acids (acetate, isobutyrate, and propionate) were not significantly different among the groups (*p* > 0.05), except for succinate levels, which were downregulated in HC/LC, HT/LC, and LT/LC. Additionally, glucose levels increased significantly under HT/LT, likely because of increased carbohydrate metabolism.

### Result of Transcriptome Analysis

#### Annotation of *Symbiodinium* sp. Transcriptome

**Figure 4:**
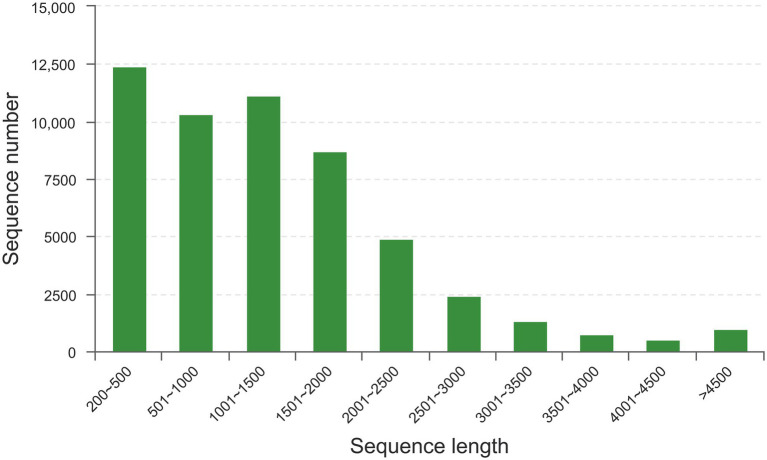
The length distribution of unigenes. X-axis, sequence length; Y-axis, sequence number for corresponding sequence length.

To investigate the potential effect of taurine on carbon metabolism in *Symbiodinium* sp., we analyzed the transcriptome of *Symbiodinium* sp. exposed to 1 mM taurine (HT, with HC as control). In total, an average of 58,630,949 raw reads and 58,147,131 clean reads were generated from HT and HC groups; 98.42% of the clean read bases had a value of *Q* ≥ 20, and 95.31% of the clean read bases had a value of *Q* ≥ 30 (Supplementary Table S2). *De novo* assembly generated a total of 53,545 unigenes with an average length of 1,377 bp, N50 length of 1859 bp, and E90N50 length of 1901 bp (Supplementary Table S3). Figure 4 shows the length distribution of unigenes. Details for the annotation of transcriptome were provided as Supplementary Materials (Supplementary Tables S4–S7 and Supplementary Figures S2–S5).

#### Identification and Functional Enrichment Analysis of DEGs

Taurine addition did not lead to strong changes in the transcriptome; only 208 out of 53,545 detected unigenes were varied significantly (*p* < 0.05 and fold change >2) under taurine-stimulated conditions (Supplementary Tables S8 and S9).

**Figure 5:**
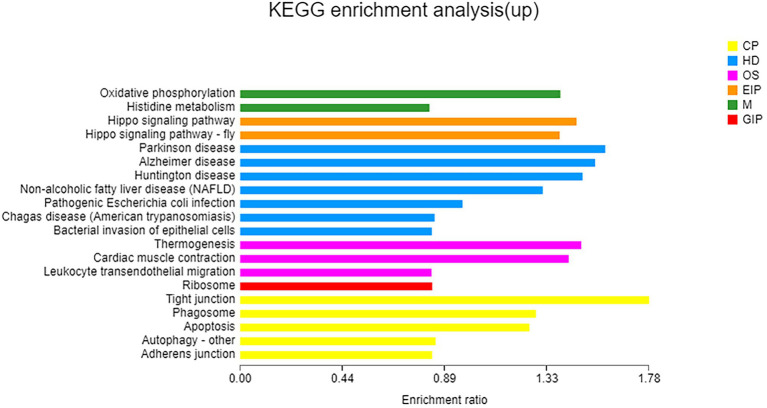
KEGG enrichment analysis of upregulated genes. X-axis, enrichment ratio; Y-axis, functional categories.

Based on COG classification, DEGs were classified into energy production and conversion; translation, ribosomal structure, and biogenesis; posttranslational modification, protein turnover, chaperones; inorganic ion transport and metabolism; function unknown; signal transduction mechanisms; and cytoskeleton (Supplementary Figure S6). GO enrichment analysis revealed that these upregulated DEGs were enriched in only one MF category: odorant binding. KEGG enrichment analysis revealed that these upregulated DEGs were enriched in oxidative phosphorylation (Figure 5). Noticeably, some genes involved in nitrogen metabolism and amino acid metabolism, including a high-affinity nitrate transporter, a phosphoserine phosphatase which catalyzes the conversion of glycerate-3P to serine, an N-acetyltransferase which catalyzes the conversion of glutamate to ornithine, and an amino acid permease, were upregulated (*p* < 0.05) under taurine-stimulated conditions (Supplementary Table S8).

**Figure 6:**
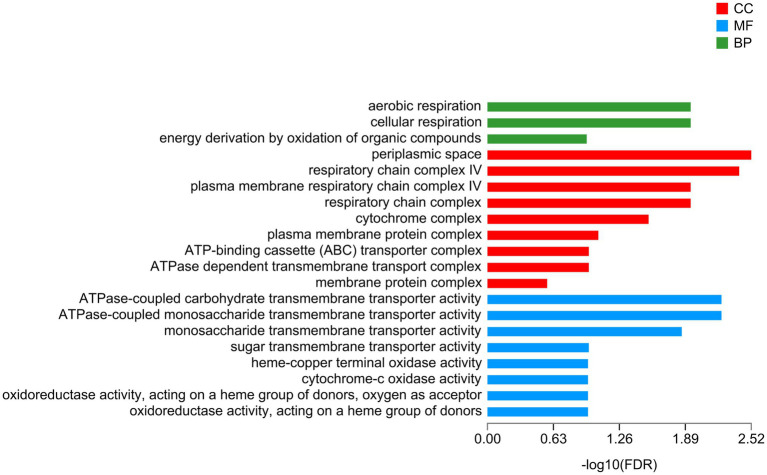
GO enrichment analysis of downregulated genes. X-axis, −log_10_ (FDR); Y-axis, functional categories.

**Figure 7:**
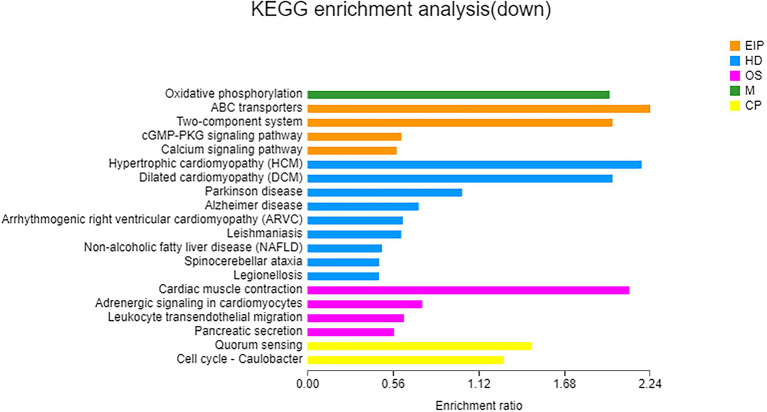
KEGG enrichment analysis of downregulated genes. X-axis, enrichment ratio; Y-axis, functional categories.

A total of 112 downregulated DEGs were identified (Supplementary Table S9), out of which 75 were annotated. Based on COG classification, they were classified into categories including amino acid transport and metabolism, carbohydrate transport and metabolism, and lipid transport and metabolism (Supplementary Figure S7). GO enrichment analysis (Figure 6) revealed that these downregulated DEGs were enriched in BP terms such as aerobic respiration; CC terms such as periplasmic space; and MF terms such as ATPase-coupled carbohydrate transmembrane transporter activity. These results highlighted the influence of taurine carbohydrate transport, organic acid transport, and amino acid transport. KEGG enrichment analysis revealed that these downregulated DEGs were enriched in oxidative phosphorylation (Figure 7).

### Differences in Cell Composition Under HC and HT

**Figure 8:**
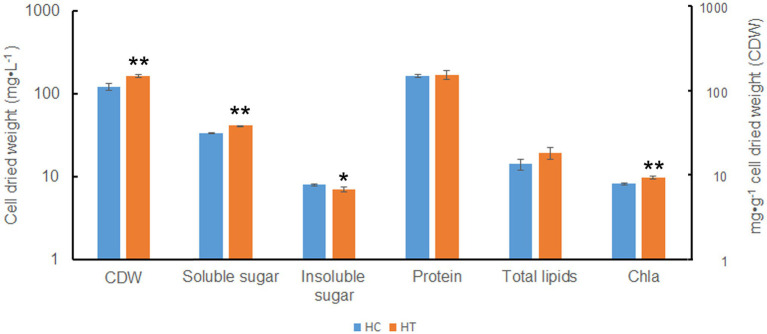
Biomass and cellular components in *Symbiodinium* sp. under HC and HT conditions. X-axis, group of cellular components; Y-axis, cell dried weight (CDW, indicated as mg·L^−1^) and cellular component content (indicated as mg·g^−1^ cell dried weight). Data points are the means of triplicates and error bars represent the standard deviation. Data points are the means of triplicates and error bars represent the standard deviation. *indicates significant differences (value of *p* <0.05), **indicates very significant differences (value of *p* <0.01) between groups evaluated by Student’s *t*-test.

Compared to HC, HT increased in cellular total soluble sugar but decreased in total insoluble sugar (value of *p* < 0.05; Figure 8). Besides, the content of Chla also increased (value of *p* < 0.05). There were no significant changes in the content of total protein and total lipids (value of *p* > 0.05). This indicated that taurine might influence the metabolism of *Symbiodinium* sp. by decreasing the content of total insoluble (most probably storage compounds) while increasing the synthesis of total soluble sugar (most probably translocated compounds), which might be further secreted into the extracellular for utilization in coral.

## Discussion

Coral reefs play important roles in the marine ecosystem (Yu, 2018). In order to protect the coral reefs, it is necessary to have an in-depth understanding of their formation processes and mechanisms. The formation of coral reefs depends to a great extent on the symbiotic relationship between coral animals and *Symbiodinium* sp. (Muscatine and Cernichiari, 1969). Photosynthates supplied by *Symbiodinium* sp. to their coral hosts can fulfill 95% of the latter’s energy requirements. Researchers had studied the interaction between coral animals and their symbiotic *Symbiodinium* sp., especially the mechanism by which corals induce the release of photosynthates from *Symbiodinium* sp. Previous studies suggested that corals control the flow of carbon fixation products in *Symbiodinium* sp. through the release of HRFs (mainly free amino acids, such as taurine; Gates et al., 1995; Wang and Douglas, 1997; Davy and Cook, 2001; Whitehead and Douglas, 2003; Grant et al., 2004). However, the form in which the photosynthates are transported and the underlying mechanism of HRF release has not been elucidated yet. In this study, based on the results of growth, cell composition analysis, metabolomics, and transcriptomic analysis, we found that taurine at a concentration of 1 mM significantly increased the growth (Figure 1), actual photosynthesis efficiency (Figure 2), biomass, total soluble sugar, chlorophyll a contents (Figure 8) in *Symbiodinium* sp. The contents of most detected free amino acids and glucose were also increased (Figure 3; Supplementary Table S1). While the content of total insoluble sugar decreased (Figure 8). This suggested that taurine tended to alter the metabolism of *Symbiodinium* sp. from synthesis of storage compounds to synthesis of translocated compounds, which might be further secreted into the extracellular for utilization in coral. This provided further evidence for the role of HRF of taurine in *Symbiodinium* sp. Increased in the content of *Chla* might be beneficial to photosynthesis, which was consistent with the results of photosynthesis parameters detection.

As for the action mechanism of HRF, two explanations exist according to previous studies. Initially, HRF was thought to induce the release of photosynthates by altering the permeability of the algal cell membrane (Furla et al., 1998; Whitehead and Douglas, 2003). Currently, increasing evidence suggests that HRF regulates the flow of carbon produced by *Symbiodinium* sp. by diverting photosynthetically fixed carbon from storage compounds to translocated compounds such as soluble sugar (Davy and Cook, 2001). To investigate the potential role of taurine in the regulation of carbon metabolism in *Symbiodinium* sp., we performed transcriptome analysis under control (HC) and taurine-stimulated (HT) conditions. A cell wall-associated hydrolase was upregulated (FC = 3.07, value of *p* = 0.0001). Besides, some genes involved in hydrolase, including a putative peptidase C1-like protein (FC = 7.84, value of *p* = 0.0142), an esterase OVCA2 (FC = 3.91, value of *p* = 0.0307), and a phosphoserine phosphatase (FC = 2.61, value of *p* = 0.0021), were upregulated. It is reported that HRFs might alter the permeability of cell membrane of symbiotic algae to facilitate the release of photosynthetic products (Furla et al., 1998; Whitehead and Douglas, 2003). In this study, the increase of cell wall-associated hydrolase and other hydrolase-related genes indicated that taurine might also act by altering the permeability of the *Symbiodinium* sp. cell membrane. Moreover, some genes involved in the metabolism of storage compounds such as proteins and lipids, including a peptidase (FC = 7.84, value of *p* = 0.0142), a putative E3 ubiquitin-protein ligase (FC = 2.90, value of *p* = 0.035), an apolipophorin (FC = 11.56, value of *p* = 0.016), and an esterase (FC = 3.91, value of *p* = 0.0307), were upregulated, indicating that taurine might induce the alteration of *Symbiodinium* sp. metabolism from synthesis of storage compounds to decomposing of them, providing new evidence for previous reports (Davy and Cook, 2001). Meanwhile, the metabolomics results indicated an increase in free amino acids content, and cell composition analysis indicated an increase in total soluble sugar content and a decrease in total insoluble sugar content, suggesting that taurine might also act by altering the metabolism of *Symbiodinium* sp. from synthesis of storage compounds to synthesis of translocated compounds. Unexpectedly, among the downregulated DEGs, 12 were involved in the transport of organic substances such as amino acids, glucose, and organic acids. This seemed to contradict to our speculate that the permeability of algal cell membrane is increased by taurine. It is probable that the transcriptional changes may be a proactive response of the *Symbiodinium* sp. to reduce the influence of host, i.e., to avoid excessive transport of photosynthetically fixed carbon from symbiotic algae to the host.

Generally, under starvation conditions, corals induce the higher production of photosynthetic products in *Symbiodinium* sp. (Wang and Douglas, 1998, 1999; Davy and Cook, 2001). During coral starvation, the levels of nitrogen decrease due to a decrease in its metabolism, thereby leading to a reduction in the availability of nitrogen for *Symbiodinium* sp. (Davy and Cook, 2001). In our study, a gene encoding a high-affinity nitrate transporter was found to be upregulated (FC = 3.38, value of *p* = 0.0235). We speculate that taurine might be a signal of low nitrogen concentration to *Symbiodinium* sp., which in turn increase in nitrogen assimilation to maintain efficient photosynthetic carbon fixation. In addition, we also found that two of the downregulated genes are related to quorum sensing (FC = 0.022, value of *p* = 0.00015; and FC = 0.075, value of *p* = 0.0210). We speculated that taurine regulates the *Symbiodinium* sp. cell density through the quorum sensing effect and maintains a certain number of *Symbiodinium* sp. in its cells. This helps to keep the corals and *Symbiodinium* sp. at a state of dynamic balance. However, the results of our study need to be validated in future more extensive studies.

In this study, we performed transcriptome analysis by taurine-stimulated for 10 days. By setting a 2-fold change in abundance and *p* < 0.05 as the criterion of biologically significant difference in the transcriptome, only 208 DEGs were identified, including 96 upregulated and 112 downregulated DEGs. This indicates that taurine has little effect on the transcriptome of *Symbiodinium* sp. after 10 days of treatment. A shorter time point might result in more results. Besides, an exometabolomics analysis would provide further evidences. Therefore, future studies are expected to analyze the role of taurine in coral-algal symbiosis in detail.

## Conclusion

Taurine significantly increased the growth, biomass, photosynthesis efficiency, total soluble sugar, chlorophyll a, and free amino acid content of *Symbiodinium* sp. while decreased the content of total insoluble sugar. Results of metabolome and transcriptome analyses revealed that taurine might affect metabolic pathways in *Symbiodinium* sp. by altering the permeability of the algal cell membrane, diverting photosynthetically fixed carbon from storage compounds to translocated compounds, releasing a signal of low nitrogen concentration to initiate a series of responses to external stimuli, and controlling *Symbiodinium* sp. density *via* the quorum sensing effect.

## Data Availability Statement

The datasets presented in this study can be found in online repositories. The names of the repository/repositories and accession number(s) can be found in the article/ Supplementary Material.

## Author Contributions

ZX designed the study. HS and RC performed the experiments. AH, HS, and XC analyzed the data. AH wrote the manuscript. All authors contributed to the article and approved the submitted version.

## Funding

This research was financially supported by the National Natural Science Foundation of China (41876158 and 42006145), Natural Science Foundation of Hainan Province (420QN219), and the Scientific Research Foundation of Hainan University [KYQD(ZR)20060].

## Conflict of Interest

The authors declare that the research was conducted in the absence of any commercial or financial relationships that could be construed as a potential conflict of interest.

## Publisher’s Note

All claims expressed in this article are solely those of the authors and do not necessarily represent those of their affiliated organizations, or those of the publisher, the editors and the reviewers. Any product that may be evaluated in this article, or claim that may be made by its manufacturer, is not guaranteed or endorsed by the publisher.

## Footnotes

^1^ https://github.com/jstjohn/SeqPrep

^2^ https://github.com/najoshi/sickle

^3^ http://deweylab.biostat.wisc.edu/rsem/

^4^ http://www.bioconductor.org/packages/2.12/bioc/html/edgeR.html

## Supplementary Material

The Supplementary Material for this article can be found online at: https://www.frontiersin.org/articles/10.3389/fmicb. 2022.797688/full#supplementary-material
