# Supplementary material for: Effects of Taurine on Primary Metabolism and Transcription in a Coral Symbiodinium sp
Source: Front Microbiol. 2022 Jul 11;13:797688. doi: 10.3389/fmicb.2022.797688 (PMC9309572; doi:10.3389/fmicb.2022.797688)
Supplement: Supplementary file 1 [file Data_Sheet_1.docx]

1. **RNA extraction**

Total RNA was extracted from the tissue using TRIzol® Reagent according the manufacturer’s instructions (Invitrogen) and genomic DNA was removed using DNase I (TaKara). Then RNA quality was determined by 2100 Bioanalyser (Agilent) and quantified using the ND-2000 (NanoDrop Technologies). Only high-quality RNA sample (OD260/280=1.8~2.2, OD260/230≥2.0, RIN≥6.5, 28S:18S≥1.0, >2μg) was used to construct sequencing library.

**2. Library preparation, and Illumina Hiseq xten/NovaSeq 6000 Sequencing**

RNA purification, reverse transcription, library construction and sequencing were performed at Shanghai Majorbio Bio-pharm Biotechnology Co., Ltd. (Shanghai, China) according to the manufacturer’s instructions (Illumina, San Diego, CA).The ***Symbiodinium* sp.** RNA-seq transcriptome libraries were prepared using Illumina TruSeqTM RNA sample preparation Kit (San Diego, CA). Poly(A) mRNA was purified from total RNA using oligo-dT-attached magnetic beads and then fragmented by fragmentation buffer. Taking these short fragments as templates, double-stranded cDNA was synthesized using a SuperScript double-stranded cDNA synthesis kit (Invitrogen, CA) with random hexamer primers (Illumina). Then the synthesized cDNA was subjected to end-repair, phosphorylation and ‘A’ base addition according to Illumina’s library construction protocol. Libraries were size selected for cDNA target fragments of 200–300 bp on 2% Low Range Ultra Agarose followed by PCR amplified using Phusion DNA polymerase (New England Biolabs, Boston, MA) for 15 PCR cycles. After quantified by TBS380, two RNAseq libraries were sequenced in single lane on an Illumina Hiseq xten/NovaSeq 6000 sequencer (Illumina, San Diego, CA) for 2×150bp paired-end reads.

**3. Analysis of Basic Student Trust**

3.1 De novo Assembly and Annotation

The raw paired end reads were trimmed and quality controlled by SeqPrep (https://github.com/jstjohn/SeqPrep ) and Sickle (https://github.com/najoshi/sickle ) with default parameters. Then clean data from the ***Symbiodinium* sp.** samples were used to do de novo assembly with Trinity (http://trinityrnaseq.sourceforge.net/) ^[1]^. All the assembled transcripts were searched against the NCBI protein nonredundant (NR), COG, and KEGG databases using BLASTX to identify the proteins that had the highest sequence similarity with the given transcripts to retrieve their function annotations and a typical cut-off E-values less than 1.0×10^−5^ was set. BLAST2GO (http://www.blast2go.com/b2ghome)^[2]^ program was used to get GO annotations of unique assembled transcripts for describing biological processes, molecular functions and cellular components. Metabolic pathway analysis was performed using the Kyoto Encyclopedia of Genes and Genomes (KEGG, [http://www.genome.jp/kegg/)](http://www.genome.jp/kegg/)[3].)^[[3]](http://www.genome.jp/kegg/)[3].)^[.](http://www.genome.jp/kegg/)[3].)

3.2 Differential expression analysis and Functional enrichment

To identify DEGs (differential expression genes) between two different samples, the expression level of each transcript was calculated according to the transcripts per million reads (TPM) method. RSEM (http://deweylab.biostat.wisc.edu/rsem/) ^[4]^ was used to quantify gene abundances. Essentially, differential expression analysis was performed using the DESeq2^[5]^/EdgeR^[6]^with Q value ≤ 0.05, DEGs with |log2FC|>1 and Q value <= 0.05(DESeq2 or EdgeR) /Q value <= 0.001(DEGseq) were considered to be significantly different expressed genes. In addition, functional-enrichment analysis including GO and KEGG were performed to identify which DEGs were significantly enriched in GO terms and metabolic pathways at Bonferroni-corrected P-value ≤0.05 compared with the whole-transcriptome background. GO functional enrichment and KEGG pathway analysis were carried out by Goatools (https://github.com/tanghaibao/Goatools) and KOBAS (http://kobas.cbi.pku.edu.cn/home.do)^[7]^.

Reference：

[1] Grabherr MG, Haas BJ, Yassour M, Levin JZ, Thompson DA, Amit I, et al. Full-length transcriptome assembly from RNA-Seq data without a reference genome. Nat Biotechnol 2011;29:644-52.

[2] Conesa A, Gotz S, Garcia-Gomez JM, Terol J, Talon M, Robles M. Blast2GO: a universal tool for annotation, visualization and analysis in functional genomics research. BIOINFORMATICS 2005;21:3674-6.

[3] Goto MKaS. KEGG: Kyoto Encyclopedia of Genes and Genomes. Nucleic Acids Research 2000;28:27-30.

[4] Li B, Dewey CN. RSEM: accurate transcript quantification from RNA-Seq data with or without a reference genome. BMC Bioinformatics 2011;12:323.(RSEM)

[5] Love M I, Huber W, Anders S. Moderated estimation of fold change and dispersion for RNA-seq data with DESeq2[J]. Genome biology, 2014, 15(12): 550.(DESeq2)

[6] Robinson M D, McCarthy D J, Smyth G K. edgeR: a Bioconductor package for differential expression analysis of digital gene expression data[J]. Bioinformatics, 2010, 26(1): 139-140.(edgeR)

[7] Xie C, Mao X, Huang J, Ding Y, Wu J, Dong S, et al. KOBAS 2.0: a web server for annotation and identification of enriched pathways and diseases. Nucleic Acids Res 2011;39:W316-22.
