# Supplementary material for: Effects of Taurine on Primary Metabolism and Transcription in a Coral Symbiodinium sp
Source: Front Microbiol. 2022 Jul 11;13:797688. doi: 10.3389/fmicb.2022.797688 (PMC9309572; doi:10.3389/fmicb.2022.797688)
Supplement: Supplementary file 2 [file Data_Sheet_2.docx]

>TAGATTAGCCATGCATGTCTCAGTATAAGCTTTTACACGGCGAAACTGCGAATGGCTCATTAAAGCAGTTATAATTTATTTGATGGTCACTGCTACATGGATAACTGTGGTAATTCTAGAGCTAATACATGCACCAAAACCCAACTTCGCAGAAGGGTTGTATTTATTAGATACAGAACCAACGCAGGCTCCGCCTGGTTGTGGTGATTCATGATAACTCGATGAATCGTGTGGCTTGGCCGACGATGCATCTTTCAAGTTTCTGACCTATCAGCTTCCGACGGTAGGGTATGGGCCTACCGTGGCAATGACGGGTAACGGAGAATTAGGGTTTGATTCCGGAGAGGGAGCCTGAGAAACGGCTACCACATCTAAGGAAGGCAGCAGGCGCGCAAATTACCCAATCCTGACACAGGGAGGTAGTGACAAGAAATAACAATACAGGGCATCCATGTCTTGTAATTGGAATGAGTAGAATTTAAACCCCTTTATGAGTATCAATTGGAGGGCAAGTCTGGTGCCAGCAGCCGCGGTAATTCCAGCTCCAATAGCGTATATTAAAGTTGTTGCGGTTAAAAAGCTCGTAGTTGGATTTCTGTTGAGGATGACCGGTCCGCCTTCTGGGTGTGCATCTGGCTCAGCCTTGACATCTTCCTAAAGAACGTATCTGCACTTCATTGTGTGGTGCGGTATTTAGGACATTTACCTTGAGGAAATTAGAGTGTTTCAAGCAAGCGATTGCCTTGAATACATTAGCATGGAATAATAAGATAGGACCTCAGTTCTATTTTGTTGGTTTCTAGAGCTGAGGTAATGGTCGATAGGGATAGTTGGGGGCATTCGTATTTAACTGTCAGAGGTGAAATTCTTGGATTTGTTAAAGACGGACTACTGCGAAAGCATTTGCCAAGGATGTTTTCATTGATCAAGAACGAAAGTTAGGGGATCGAAGACGATCAGATACCGTCCTAGTCTTAACCATAAACTATGCCAACTAGAGATTGGAGGTCGTTACTTATACGACTCCTTCAGCACCTTATGAGAAATCAAAGTCTTTGGGTTCCGGGGGGAGTATGGTCGCAAGGCTGAAACTTAAAGGAATTGACGGAAGGGCACCACCAGGAGTGGAGCCTGCGGCTTAATTTGACTCAACACGGGGAAACTTACCAGGTCCAGACATAGTAAGGATTGACAGATTGATAGCTCTTTCTTGATTCTATGGGTGGTGGTGCATGGCCGTTCTTAGTTGGTGGAGTGATTTGTCTGGTTAATTCCGTTAACGAACGAGACCTTAACCTGCTAAATAGTTACATGTAACCTCGGTTACATGGGCAACTTCTTAGAGGGACTTTGTGTGTCTAACGCAAGGAAGTTTGAGGCAATAACAGGTCTGTGATGCCCTTAGATGTCCTGGGCTGCACGCGCGCTACACTGATGCGCTCAACGAGTTTGCAATCTTGCCTGAAATGGCCGGGTAATCTTTTTAAAATGCATCGTGATGGGGATAGATCATTGCAATTATTGATCTTCAACGAGGAATTCCTAGTAAGCGCGAGTCATCAGCTCGTGCTGATTACGTCCCTGCCCTTTGTACACACCGCCCGTCGCTCCTACCGATTGAGTGATCCGGTGAATTATTCGGACTGACGCAGTGCTCAGCTTCTGGACGTTGCGTTGGAAAGTTTCATGAACCTTATCACTAGAGGAAGGAGAAGTCGAGCATG
