# Supplementary figures and images for: Effects of Taurine on Primary Metabolism and Transcription in a Coral Symbiodinium sp
Source: Front Microbiol. 2022 Jul 11;13:797688. doi: 10.3389/fmicb.2022.797688 (PMC9309572; doi:10.3389/fmicb.2022.797688)

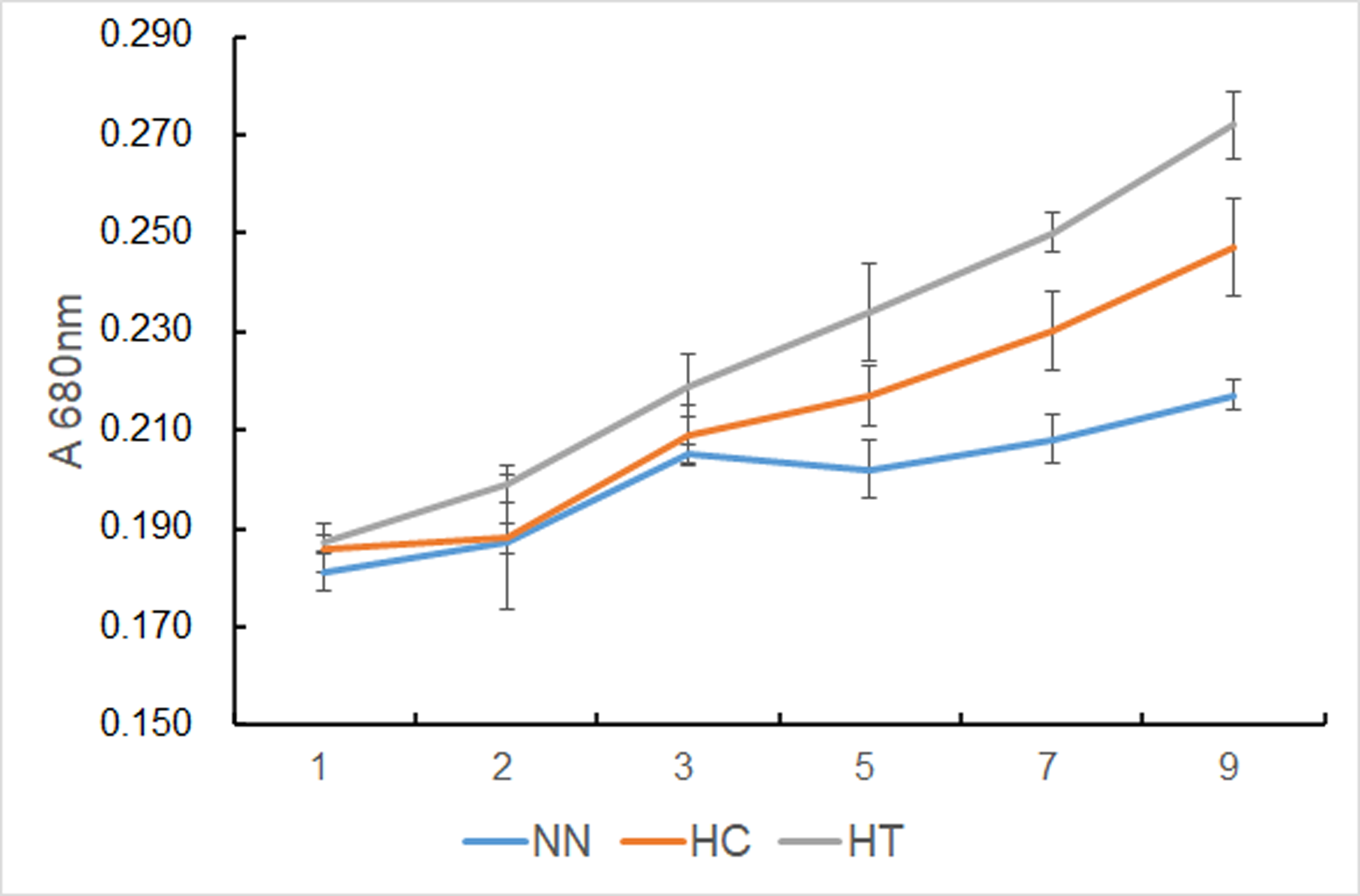

Supplement: Supplementary file 3 [file Image_1.tif]

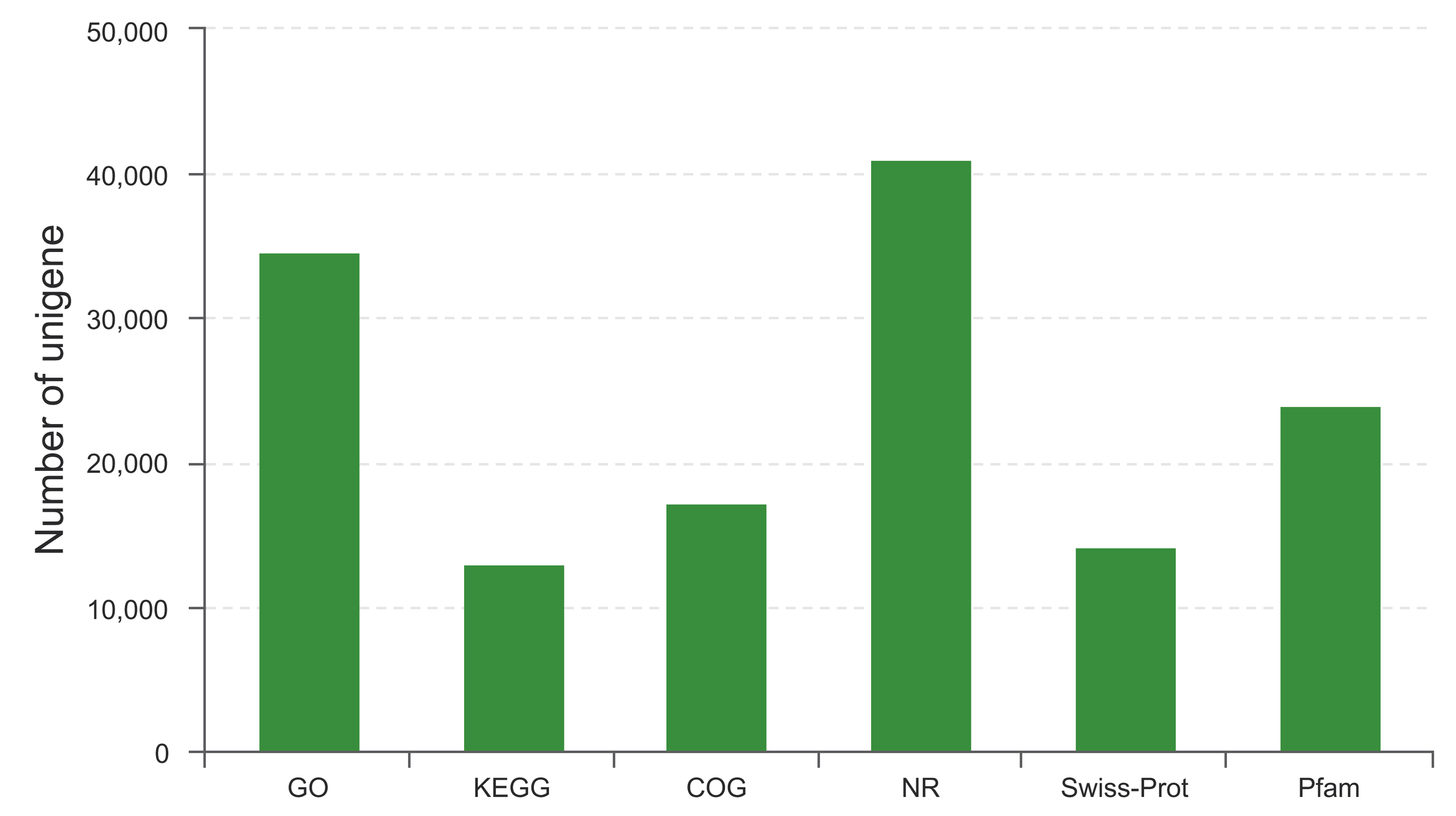

Supplement: Supplementary file 4 [file Image_2.tif]

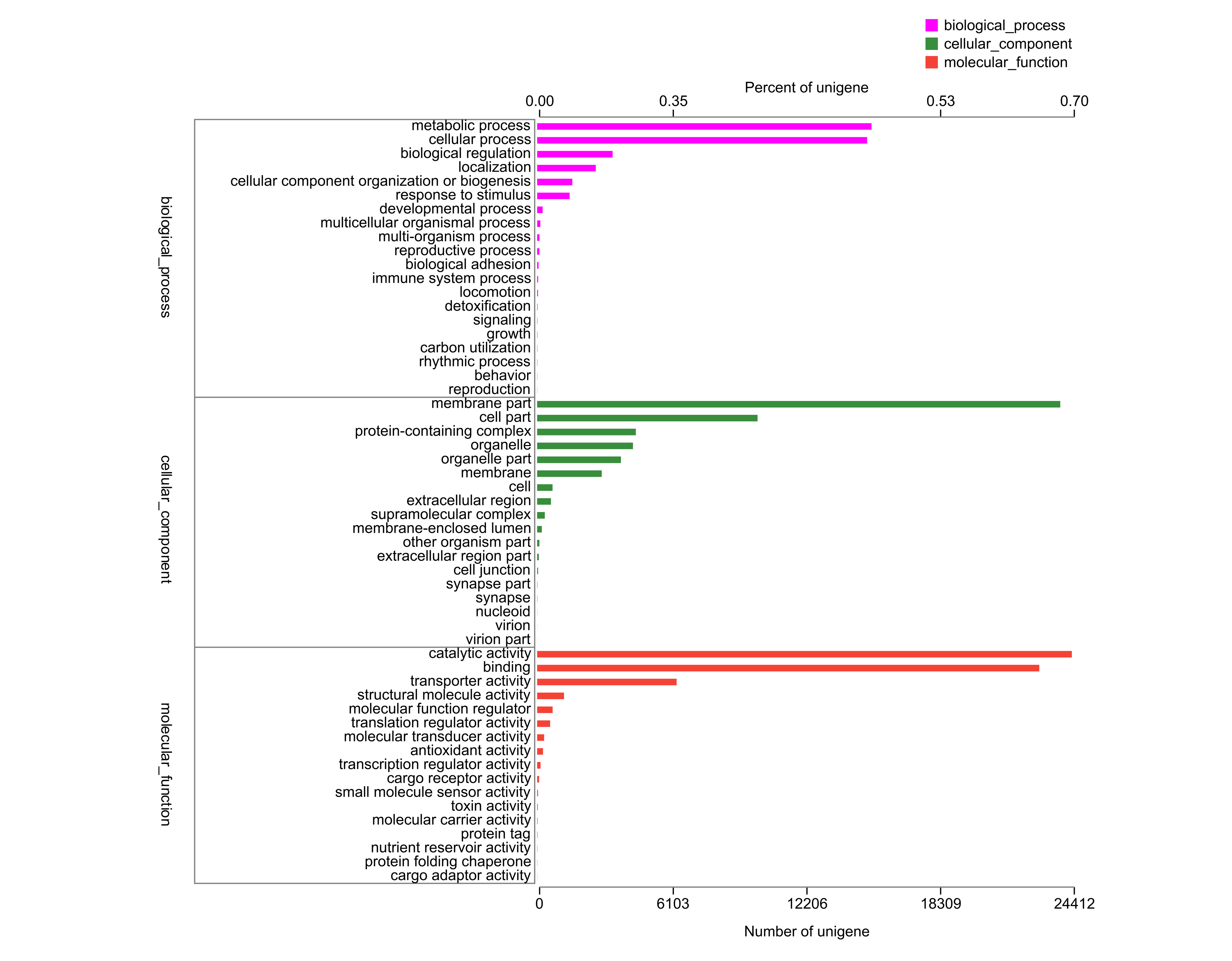

Supplement: Supplementary file 5 [file Image_3.tif]

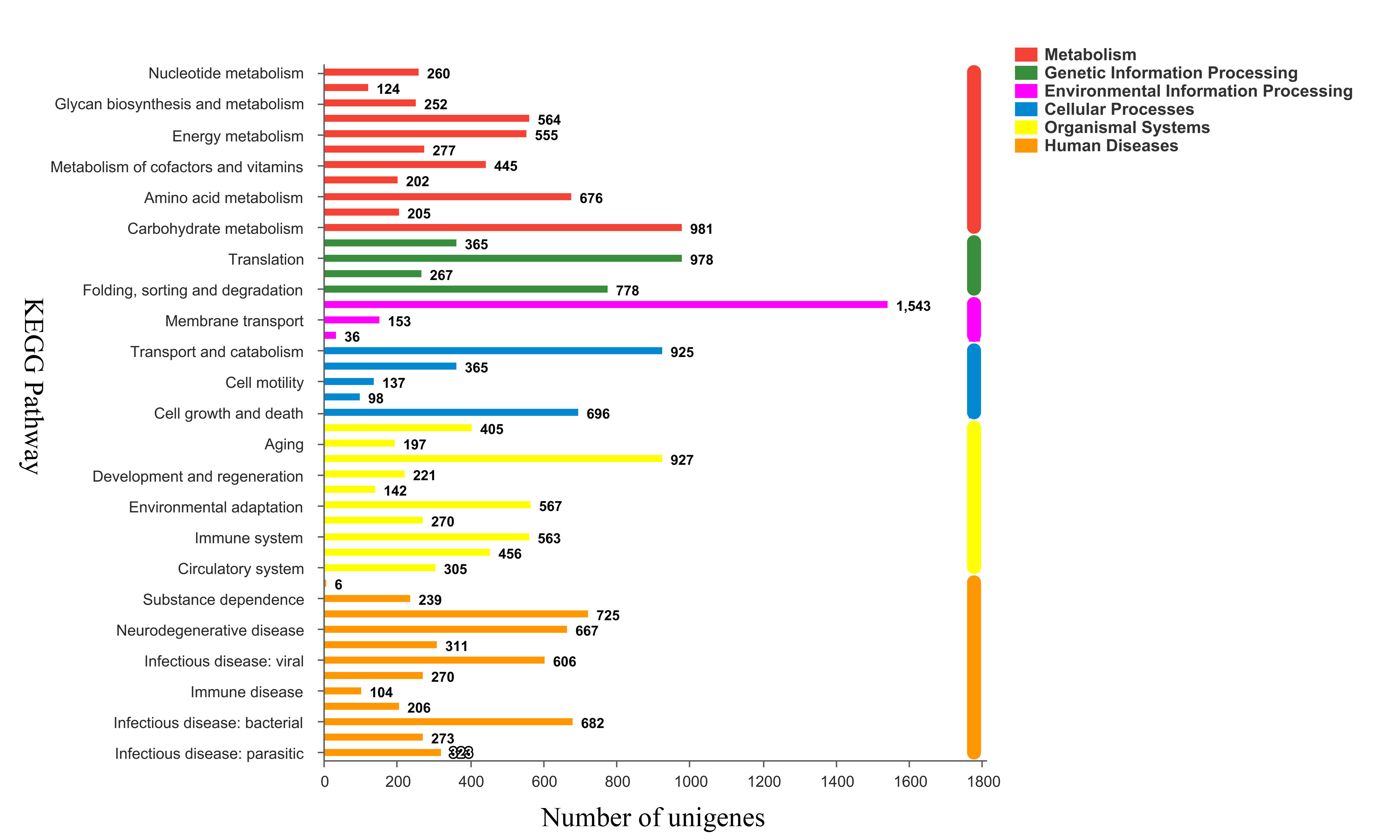

Supplement: Supplementary file 6 [file Image_4.tif]

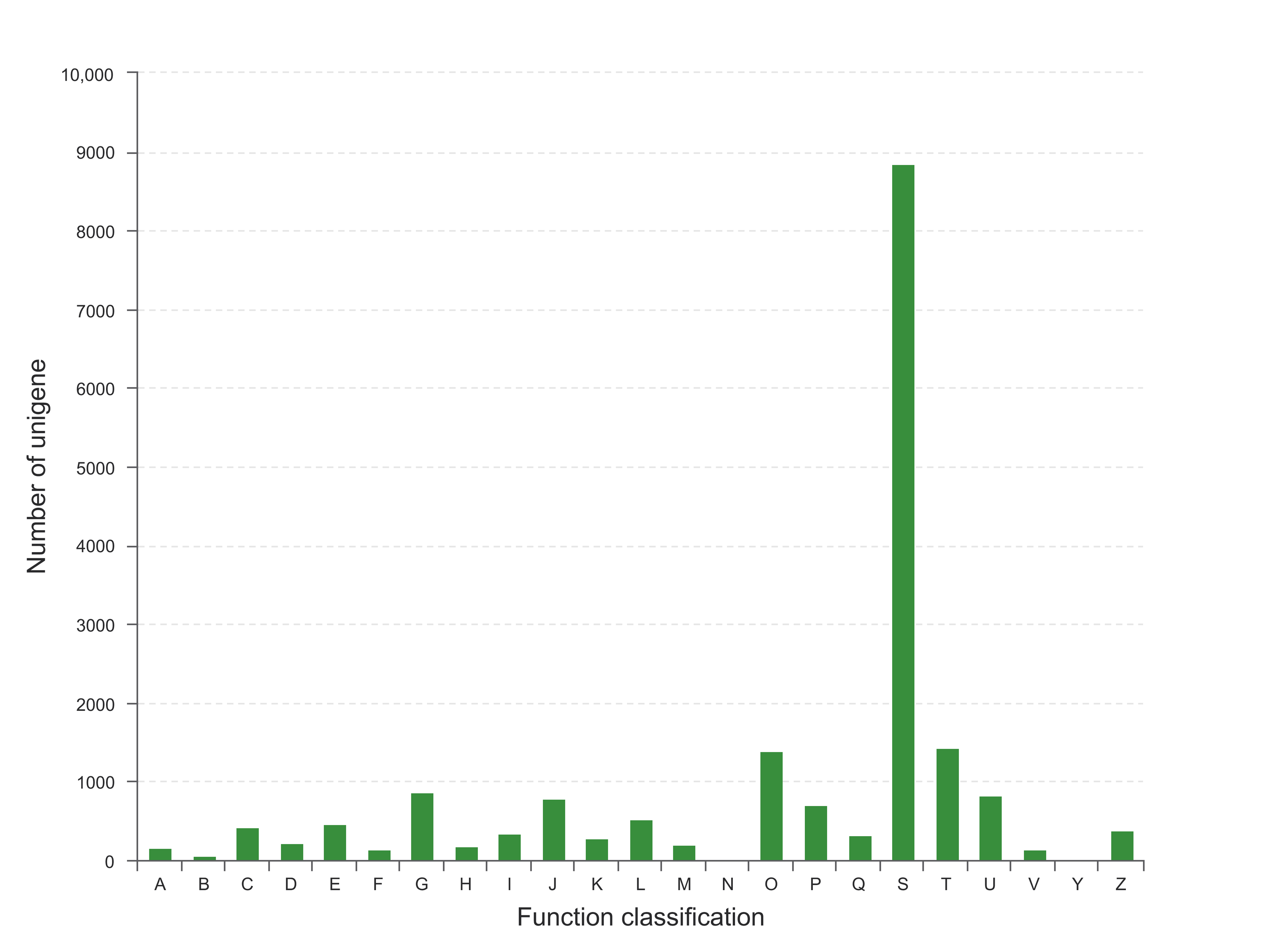

Supplement: Supplementary file 7 [file Image_5.tif]

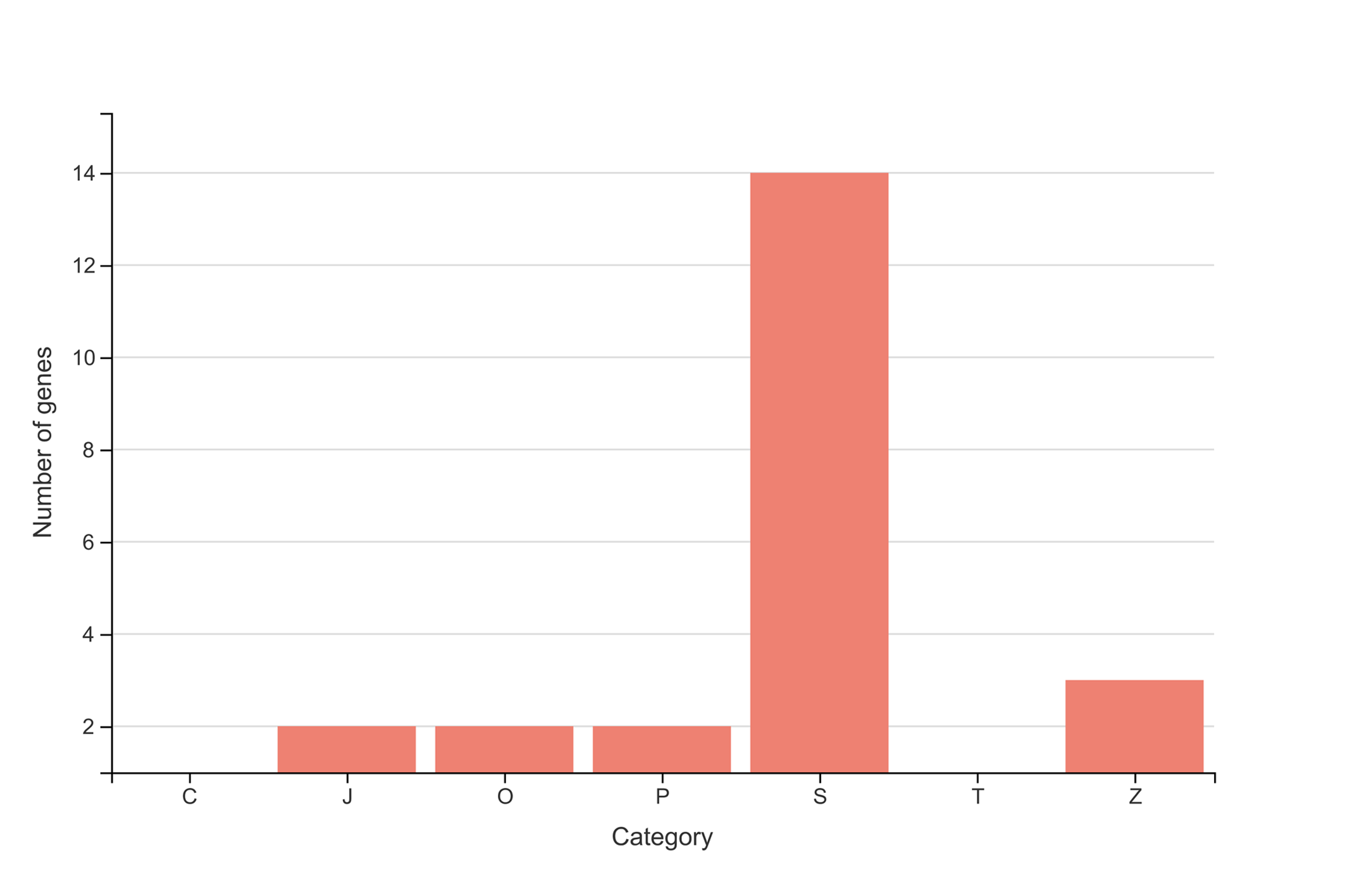

Supplement: Supplementary file 8 [file Image_6.tif]

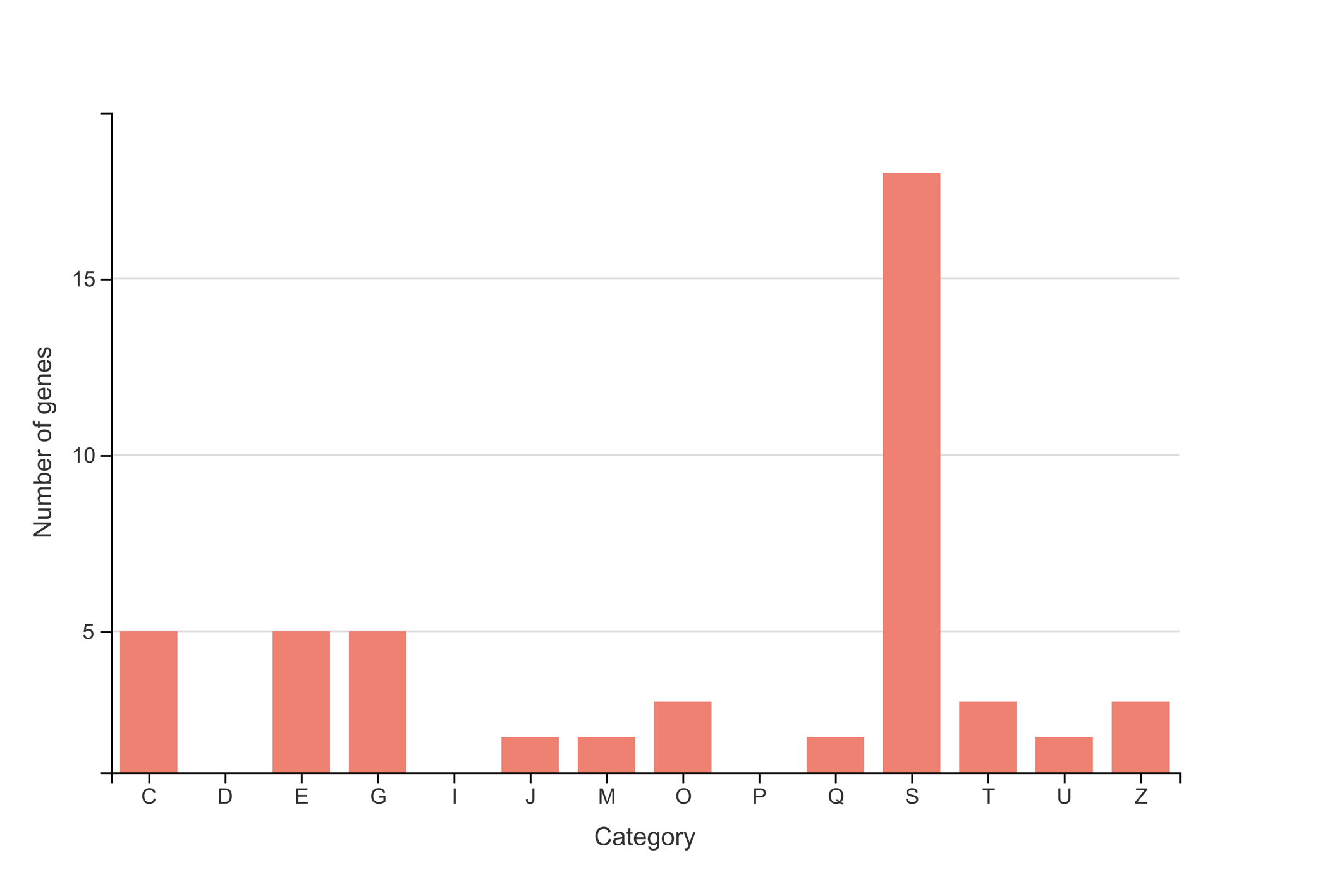

Supplement: Supplementary file 9 [file Image_7.tif]
