# Supplementary material for: Effects of Taurine on Primary Metabolism and Transcription in a Coral Symbiodinium sp
Source: Front Microbiol. 2022 Jul 11;13:797688. doi: 10.3389/fmicb.2022.797688 (PMC9309572; doi:10.3389/fmicb.2022.797688)
Supplement: Supplementary file 10 [file Table_1.docx]

**Table 1. The ratios of fold changes in key cellular metabolites detected by NMR.**

* indicates significant differences (P-value < 0.05), ** indicates very significant differences (P-value < 0.01)**.**

| group | metabolites | HC/LC | HT/HC | HT/LT | LT/LC | HT/LC | HC/LT |
| --- | --- | --- | --- | --- | --- | --- | --- |
| amines | Trimethylamine | 1.011 | 1.276 | 1.301 | 0.991 | 1.289 | 1.020 |
| amino acids | Alanine | 0.882 | 1.507^*^ | 1.536^**^ | 0.866^*^ | 1.330^*^ | 1.019 |
| amino acids | Arginine | 1.851^*^ | 1.565 | 2.350^**^ | 1.232 | 2.896^**^ | 1.502 |
| amino acids | Betaine | 0.953 | 1.466* | 1.509* | 0.926 | 1.396* | 1.029 |
| amino acids | Carnitine | 0.976 | 1.148 | 1.153 | 0.972 | 1.120 | 1.004 |
| amino acids | Glutamate | 1.048 | 1.415* | 1.438** | 1.031 | 1.483** | 1.016 |
| amino acids | Glutamine | 1.211 | 1.370* | 1.384* | 1.199 | 1.659** | 1.010 |
| amino acids | Glycine | 1.382 | 0.787 | 1.022 | 1.065 | 1.088 | 1.298 |
| amino acids | Isoleucine | 0.888 | 1.235 | 1.260 | 0.870 | 1.097 | 1.021 |
| amino acids | Leucine | 0.883 | 1.311 | 1.421* | 0.815 | 1.158 | 1.084 |
| amino acids | Lysine | 1.406* | 1.020 | 0.976 | 1.469** | 1.434** | 0.957 |
| amino acids | O-Acetylcarnitine | 0.963 | 0.939 | 0.894 | 1.010 | 0.904 | 0.953 |
| amino acids | Ornithine | 1.280 | 1.504* | 1.532** | 1.257 | 1.925** | 1.019 |
| amino acids | Threonine | 1.005 | 1.322 | 1.413** | 0.940 | 1.328* | 1.069 |
| amino acids | Tryptophan | 1.015 | 1.130 | 1.208 | 0.949 | 1.147 | 1.069 |
| amino acids | Tyrosine | 0.673* | 1.660** | 1.617** | 0.691* | 1.117 | 0.974 |
| amino acids | Valine | 0.890 | 1.429* | 1.482* | 0.858* | 1.272 | 1.038 |
| food and drug compounds | 1,3-Dimethylurate | 1.076 | 1.348 | 1.445* | 1.004 | 1.451* | 1.072 |
| food and drug compounds | Trigonelline | 1.216 | 0.919 | 0.870 | 1.284* | 1.117 | 0.947 |
| nucleic acid components | Uridine | 1.268 | 1.026 | 1.545** | 0.842 | 1.301* | 1.505 |
| organic acids | Acetate | 0.901 | 0.791 | 0.881 | 0.809 | 0.713 | 1.114 |
| organic acids | Fumarate | 1.399 | 0.222* | 0.467 | 1.251 | 0.000 | 1.117 |
| organic acids | Isobutyrate | 1.211 | 0.878 | 1.154 | 0.875 | 1.098 | 1.314 |
| organic acids | Propionate | 1.061 | 0.810 | 1.002 | 0.858 | 0.859 | 1.238 |
| organic acids | Succinate | 0.729* | 1.167 | 1.122 | 0.758** | 0.851* | 0.961 |
| sugars | Glucose | 1.109 | 1.173 | 2.049** | 0.635* | 1.301 | 1.746** |
| vitamin/cofactors | Pantothenate | 0.903 | 1.115 | 1.092 | 0.922 | 1.007 | 0.979 |
